# Miscarriage and Death Resulting from Inflammation and Suppuration

**Published:** 1880-04

**Authors:** 


MISCARRIAGE AND DEATH RESULTING FROM INFLAMMATION AND SUPPURATION CAUSED BY THE PRESENCE OF MEDICINAL SUBSTANCES IMBEDDED IN THE WALLS OF THE APPENDIX VERMIFORMIS.

Mrs. B., entered the hospital July 27th, complaining of uterine pains, threatened abortion, and pain in the left hypochondriac space. It was supposed she had suffered from injury. The treatment, which was only palliative, to prevent the threatened miscarriage, consisted in the most part in enjoining rest and giving opiates. She was delivered of a five months' fœtus within three days after her admission, and died very suddenly and unexpectedly in 14 hours afterwards.

Inflammation of the omentum and intestines was found to exist, and a large quantity of purulent fluid in the abdominal cavity, with deposits of plastic lymph. The purulent fluid was found to proceed from an abscess existing in the walls of the appendix vermiformis at the point where she had complained of pain upon admission.

Five or six hardened pills (none of which were administered in this hospital) were found imbedded in the walls of the appendix, and the irritation caused by these seemed to furnish the only plausible explanation of the ulcerative inflammation which produced her death. There were no marks of injury external nor internal.—*North Carolina Medical Journal, Dec., 1879.*

